# Autism Early Intervention Providers: Their Priorities, Use of Empirically Supported Practices, and Professional Development Needs

**DOI:** 10.1007/s10803-025-06808-w

**Published:** 2025-04-03

**Authors:** Sarah Luskin-Saxby, Melanie J. Zimmer-Gembeck, Rhylee Sulek, Jessica Paynter

**Affiliations:** 1 School of Applied Psychology, Griffith University, Southport, QLD 4215 Australia; 2 Griffith Centre for Mental Health, Griffith University, Southport, QLD 4215 Australia; 3 Telethon Kids Institute, Nedlands, Australia; 4 Griffith Institute for Educational Research, Griffith University, Southport, QLD 4215 Australia

**Keywords:** Autism spectrum disorders, Early intervention, Evidence-based practice, Professional development, Research-to-practice gap

## Abstract

**Purpose:**

Autism early intervention research has indicated a research-to-practice gap, including continued use of practices with inadequate research support, and insufficient use of empirically supported practices. The present study explored the processes and mechanisms through which providers working with young children on the autism spectrum learn, select, and implement the various practices in their clinical repertoires. We addressed the role of providers’ priorities, competence, and experience with (and needs for) professional development (PD), as well as whether, in clinical practice, a provider selects for implementation interventions based on domains.

**Method:**

Providers (*n* = 136) responded to an online survey to report the interventions they used, their outcome domain priorities, confidence, and their desire for PD.

**Results:**

The most commonly used interventions were reinforcement, modeling, prompting, and visual supports, which are all supported by research evidence and classified as empirically supported practices. While most providers reported using empirically supported practices, many also used unsupported practices, especially in the sensory domain. Providers’ top priority domains for intervention were communication, challenging behavior, adaptive behavior, and social skills. Provider confidence regarding the evidence-base of the practices they used was domain-specific and related to provider priorities. Providers reported interest in PD in all empirically supported practices and in all domains.

**Conclusion:**

Results may inform the delivery of PD in early intervention services for providers working with children on the autism spectrum, to support the best possible outcomes for this population and mitigate the research-to-practice gap.

**Supplementary Information:**

The online version contains supplementary material available at 10.1007/s10803-025-06808-w.

## Introduction

Best practice guidelines for children on the autism spectrum^1^ emphasize the use of the evidence-based practice (EBP) framework to help guide decision making (Whitehouse et al., 2020; Steinbrenner et al., 2020). The EBP framework includes consideration of the best available research evidence and the practitioner’s expertise, in addition to client’s strengths, needs, values, and preferences to guide the selection of practices to implement (Sackett et al., 1996). Practices may be classified as either comprehensive treatment models (e.g., Denver Model, Rogers et al., 2012), those which involve a combination of practices which aim to target a broader range of skills, or focused intervention practices (FIPs, e.g., prompting) which typically address a specific goal or need (Odom et al., 2010). Systematic reviews, such as Wong et al. (2015) have identified FIPs that focus on specific outcome domains (i.e., adaptive behavior, challenging behavior, communication, joint attention, motor, play, pre-academic, school transition, sensory, and social skills). However, despite the identification of empirically supported FIPs, practices lacking empirical support of their efficacy (*unsupported practices*) continue to be used (Brock et al., 2014; Dynia et al., 2020; Paynter et al., 2017, 2018). Unsupported practices can lead to harm to children, false hope for families, and financial and opportunity costs (Stahmer et al., 2005; Paynter et al., 2017, 2022). The reasons behind continued use of unsupported practices by practitioners are multifactorial, including lack of knowledge and insufficient training (Dillenburger et al., 2016; Elsabbagh et al., 2014). However, there is scarce research summarizing how early intervention providers (e.g., teachers, allied health professionals, and early childhood professionals) use unsupported practices based on the *outcome domain*. Outcome domains are important to consider when selecting interventions for children on the autism spectrum, as FIPs are not applicable to every domain. Additionally, different domains may be prioritized at different developmental stages (e.g., early intervention vs. school-age), and there is variation in the amount and quality of evidence across domains ([NCAEP]; Steinbrenner et al., 2020). There is also limited information about how providers’ priorities and their confidence in their practices may influence their choice of interventions. Such findings could help encourage providers to seek the knowledge and training necessary to make informed decisions that promote the best outcomes for each outcome domain for children. The aim of this study was to understand the mechanisms by which providers working with young children on the autism spectrum learn about, select, and implement various practices based on outcome domains.

## Early Intervention Providers’ Practice Preferences and Decisions

### Use of Empirically Vs. Non-Empirically Supported Practices

To understand practice preferences and decisions of autism early intervention service providers, one issue to consider is whether practitioners can identify empirically supported practices, and whether this competence varies across outcome domains. There is some evidence that identifying practices with solid empirical support can be difficult for providers regardless of the domain. This circumstance may partly explain use of unsupported practices, either in place of or in addition to empirically supported practices (Luskin-Saxby et al., 2023; Paynter & Keen, 2015; Stahmer et al., 2005). Dynia et al. (2020) surveyed 45 U.S.A. preschool teachers working with young children on the autism spectrum about their use of practices. Teachers’ open-ended responses regarding their approaches were coded to determine if they reflected the use of empirically supported practices. Nearly all participants reported using at least one empirically supported practice (most commonly visual supports, modeling, prompting, reinforcement, and social narratives). At the same time, however, nearly half reported using unsupported practices, with these frequently targeting the sensory domain. Similarly, Luskin-Saxby et al. (2023) found a combination of supported and unsupported practices in semi-structured interviews with 15 providers across three autism early intervention services in Australia. Specifically, while the majority were using mostly practices supported by research evidence, they also reported using emerging practices (i.e., those with limited empirical support that is below the threshold to qualify them as empirically supported) and unsupported ones. The unsupported practices cited most often targeted the sensory domain and included techniques such as weighted vests (Luskin-Saxby et al., 2023).

### Providers’ Beliefs about Practice Effectiveness

Providers’ beliefs about the empirical support for practices might also influence their practice decisions and prioritization of children’s needs (Johnson et al., 2018). Paynter et al. (2019) found that perceived evidence, including erroneously rating an unsupported practice as having high empirical support, was a statistically significant predictor of early intervention providers’ intended future use of both empirically supported and unsupported practices. These findings corroborated research based on pre-service teachers’ ratings of general teaching practices (Carter et al., 2015) and were consistent with evidence that teachers make more use of practices when they rate them as more empirically supported (Dynia et al., 2020). Thus, the *belief* that a practice is empirically supported– whether accurate or not– may be an important factor impacting its adoption. Of special relevance to the present research is that such beliefs have been linked to a need for (or lack of) PD (Dillenburger et al., 2016; Elsabbagh et al., 2014).

### Practice Priorities and Confidence

Early intervention providers are tasked with supporting children on the autism spectrum across many outcome domains. Given the variety of these domains, providers’ beliefs about which ones hold higher priority may impact on their practice preferences, decisions, and feelings of confidence in their practice. Yet, in a study that identified 27 focused intervention practices and 12 outcome domains as important factors in selecting interventions for children on the autism spectrum (Wong et al., 2015), the outcome domains most commonly targeted had only a tenuous connection to the selection of specific practices. This is important, as not all practices are applicable for each outcome domain. To date, very few studies have investigated practice use according to the domain. A notable exception is Dynia et al. (2020), who asked participants to prioritize children’s needs that might be addressed when delivering supports, and to report their confidence in targeting the respective outcome domains, interest in PD, instructional approaches, and beliefs about empirically supported practice use. In that study, the top priority outcome domains were social skills, communication, and challenging behavior. Similarly, in a study of 99 special education and general education teachers (Brock et al., 2014), participants prioritized the outcome domains of social skills, communication, and pre-academic skills. Limited information is available on how Australian early intervention providers for children on the autism spectrum prioritize outcome domains, on their beliefs about the evidence base supporting the practices they use within domains, or on their confidence in providing services in each domain. The current study was designed to fill this gap.

### Professional Development and Use of Empirically Supported Practices

PD opportunities have been invaluable in encouraging the use of empirically supported practices (e.g., Luskin-Saxby et al., 2023). Conversely, a lack of PD, inadequate PD, and insufficient support for implementation of autism early interventions with fidelity have been identified as important, modifiable factors impacting the uptake of empirically supported practices (Dillenburger et al., 2016; Elsabbagh et al., 2014). Research has shown, however, that PD is often not consistently available or implemented. One study demonstrated that several early intervention centers had adopted ad hoc approaches to PD, meaning the initiatives implemented lacked planning or preparation (Luskin-Saxby et al., 2023); other studies have also found that PD offered in early intervention centers may be inadequate or insufficient (e.g., Paynter et al., 2017), with paraprofessionals frequently receiving their training from other staff or colleagues (Giangreco et al., 2001; Nail-Chiwetalu & Ratner, 2007). Two of these studies (Paynter et al., 2017; Paynter & Keen, 2015) documented that providers regularly relied on colleagues or PD provided internally by the organization to inform their clinical decision-making, rather than on research evidence, reviews, or practice guidelines. Professional staff are often responsible for selecting and implementing training for paraprofessionals working with young children on the autism spectrum (who, overall, are likely to spend more time than professionals in direct contact with children). These providers tend to rely on colleagues’ knowledge and skills, believing that that the latter would promote only empirically supported practices and train for their application with fidelity (Luskin-Saxby et al., 2023). However, senior members of multidisciplinary teams who usually upskill paraprofessionals may, themselves, not have received appropriate training to implement practices with fidelity, or may not have been taught to provide effective coaching to that effect (Giangreco et al., 2004). Such a reliance on anecdotal sources of PD may thus exacerbate the gaps in knowledge or false beliefs about the evidence base of different intervention practices (e.g., Kadar et al., 2012; Miller et al., 2012; Paynter et al., 2018).

To address these gaps, research has endeavored to identify the needs for PD in early autism intervention and the topics to cover. Brock et al. (2014) surveyed 456 teachers and administrators in the U.S.A. regarding the PD needs of staff working with students on the autism spectrum and found their interest in accessing PD was modest. Also, participants were generally not confident in their ability to implement many of the empirically supported practices or address training topics germane to their students on the autism spectrum. However, those who expressed less confidence did not necessarily show more interest in PD. These findings are consistent with research that has found that teachers’ reports of using empirically supported practices were not significantly linked to their interest in PD (Dynia et al., 2020).

## Current Study

The current study explored the practice knowledge, preferences, and priorities of early intervention providers in Australia working with young children on the autism spectrum across 10 child outcome domains and within each specific domain. The focus was on FIPs, excluding comprehensive treatment models, which aligns with previous research (e.g., Paynter et al., 2022) and the fact that practitioners typically have an option to select FIPs within their scope of practice. In contrast, comprehensive treatment models are more likely to be adopted center-wide and may be influenced by a variety of factors (e.g., funding, organizational policies). Providers also reported their experience with and preferences for PD. It should be noted that the focus is solely on the Australian context, due to the cross-country differences in funding rules and policies, training of professionals and paraprofessionals, and ethical and regulatory rules for personnel. We addressed the following research questions:

1. What percentage of participating autism early intervention providers report using unsupported practices?
2. How do autism early intervention providers perceive the evidence base of the practices they use, and does this differ across outcome domains?
3. How do autism early intervention providers prioritize potential outcome domains for early intervention? Does their confidence differ according to the domain?
4. What training and development do autism early intervention providers currently receive (e.g., online, face-to-face)? Does the training they receive focus on practices with evidence of effectiveness, while also discouraging those unsupported by evidence? What are participants’ preferences regarding who provides PD and training?
5. What are the barriers and facilitators to engaging in training and PD for autism early intervention providers?

### Method

#### Participants

The participants were 136 Australian early intervention providers working directly with children on the autism spectrum (Table 1). The majority were female (94.9%), between the ages of 26 and 35 years (34.6%) with a postgraduate qualification (55.1%); 19 (24.3%) were psychologists, 32 (42.7%) behavior analysts or therapists, 12 (14.7%) speech pathologists, and two (2.7%) were occupational therapists. Twenty participants (12.8%) reported having a disability-specific qualification (e.g., Bachelor of Special Education, Masters in Autism Studies etc.). About three-fourths of participants were from two states (QLD 44.9%, NSW 30.1%). Time in profession ranged between 1 month and 44.8 years, and time in current role between 1 months and 44.8 years. Time working with individuals on the autism spectrum ranged between 2 months and 44.8 years, with 56 (41.2%) reporting having personal experience (e.g., family member) with autism.

**Table 1:** Participant demographics by occupation

	Behavior analyst	Behavior therapist	Educator or learning facilitator	Occupational therapist	Psychologist	Social worker	Speech pathologist	Teacher	Other/Not advised	Total
	(*n* = 27)	(*n* = 14)	(*n* = 21)	(*n* = 14)	(*n* = 22)	(*n* = 2)	(*n* = 18)	(*n* = 6)	(*n* = 12)	(*n* = 136)
**Gender**										
Male	1 (3.7%)	0 (0%)	0 (0%)	0 (0%)	3 (13.4%)	0 (0%)	0 (0%)	1 (16.7%)	1 (8.3%)	6 (4.4%)
Female	26 (96.3%)	14 (100%)	21 (100%)	13 (92.9%)	19 (86.6%)	2 (100%)	18 (100%)	5 (83.3%)	11 (91.7%)	129 (94.9%)
Non-binary	0 (0%)	0 (0%)	0 (0%)	1 (7.1%)	0 (0%)	0 (0%)	0 (0%)	0 (0%)	0 (0%)	1 (0.7%)
**Age bracket**										
Under 25	1 (3.7%)	8 (57.1%)	5 (23.8%)	4 (28.6%)	3 (16.1%)	1 (50%)	6 (33.3%)	0 (0%)	2 (16.7%)	30 (22.1%)
26–35	14 (51.9%)	5 (35.7%)	3 (14.3%)	5 (35.7%)	8 (36.6%)	0 (0%)	6 (33.3%)	3 (50%)	3 (25%)	47 (34.6%)
36–45	7 (25.9%)	0 (0%)	4 (19%)	3 (21.4%)	4 (17%)	1 (50%)	4 (22.2%)	1 (16.7%)	3 (25%)	27 (19.9%)
46–55	3 (11.1%)	0 (0%)	5 (23.8%)	2 (14.3%)	6 (26.8%)	0 (0%)	1 (5.6%)	2 (33.3%)	3 (25%)	22 (16.2%)
56–65	2 (7.4%)	0 (0%)	4 (19%)	0 (0%)	0 (0%)	0 (0%)	1 (5.6%)	0 (0%)	1 (8.3%)	8 (5.9%)
66 and over	0 (0%)	1 (7.1%)	0 (0%)	0 (0%)	1 (3.6%)	0 (0%)	0 (0%)	0 (0%)	0 (0%)	2 (1.5%)
**Highest academic qualifications**										
Did not complete High School	0 (0%)	0 (0%)	0 (0%)	0 (0%)	0 (0%)	0 (0%)	0 (0%)	0 (0%)	0 (0%)	0 (0%)
Senior Certificate or equivalent	0 (0%)	3 (21.4%)	4 (19%)	0 (0%)	0 (0%)	0 (0%)	0 (0%)	0 (0%)	0 (0%)	7 (5.1%)
Diploma or equivalent	0 (0%)	0 (0%)	11 (52.4%)	0 (0%)	0 (0%)	0 (0%)	0 (0%)	2 (33.3%)	1 (8.3%)	14 (10.3%)
Bachelor Degree	0 (0%)	5 (35.7%)	3 (14.3%)	11 (78.6%)	2 (12.5%)	2 (100%)	7 (38.9%)	2 (33.3%)	5 (41.7%)	37 (27.2%)
Postgraduate degree	26 (96.3%)	6 (42.9%)	3 (14.3%)	2 (14.3%)	19 (83.9%)	0 (0%)	11 (61.1%)	2 (33.3%)	6 (50%)	75 (55.1%)
Other	1 (3.7%)	0 (0%)	0 (0%)	1 (7.1%)	1 (3.6%)	0 (0%)	0 (0%)	0 (0%)	0 (0%)	3 (2.2%)
**Location of training**										
In Australia	13 (48.1%)	12 (85.7%)	19 (90.5%)	12 (85.7%)	19 (89.3%)	1 (50%)	15 (83.3%)	5 (83.3%)	11 (91.7%)	107 (78.7%)
Overseas	6 (22.2%)	2 (14.3%)	1 (4.8%)	1 (7.1%)	1 (3.6%)	0 (0%)	2 (11.1%)	0 (0%)	0 (0%)	13 (9.6%)
Both	8 (29.6%)	0 (0%)	1 (4.8%)	1 (7.1%)	2 (7.1%)	1 (50%)	1 (5.6%)	1 (16.7%)	1 (8.3%)	16 (11.8%)
**Personal experience with autism**										
Yes	10 (37%)	5 (35.7%)	12 (57.1%)	8 (57.1%)	6 (26.8%)	1 (50%)	8 (44.4%)	0 (0%)	6 (50%)	56 (41.2%)
No	17 (63%)	9 (64.3%)	9 (42.9%)	6 (42.9%)	16 (73.2%)	1 (50%)	10 (55.6%)	6 (100%)	6 (50%)	80 (58.8%)
**Work location**										
New South Wales	10 (37%)	9 (64.3%)	2 (9.5%)	1 (7.1%)	11 (55.4%)	1 (50%)	3 (16.7%)	2 (33.3%)	2 (16.7%)	41 (30.1%)
Queensland	6 (22.2%)	2 (14.3%)	18 (85.7%)	8 (57.1%)	9 (37.5%)	0 (0%)	8 (44.4%)	3 (50%)	7 (58.3%)	61 (44.9%)
South Australia	2 (7.4%)	0 (0%)	1 (4.8%)	1 (7.1%)	0 (0%)	1 (50%)	1 (5.6%)	0 (0%)	1 (8.3%)	7 (5.1%)
Tasmania	0 (0%)	0 (0%)	0 (0%)	0 (0%)	0 (0%)	0 (0%)	0 (0%)	1 (16.7%)	0 (0%)	1 (0.7%)
Victoria	6 (22.2%)	2 (14.3%)	0 (0%)	1 (7.1%)	2 (7.1%)	0 (0%)	4 (22.2%)	0 (0%)	1 (8.3%)	16 (11.8%)
Western Australia	3 (11.1%)	0 (0%)	0 (0%)	3 (21.4%)	0 (0%)	0 (0%)	2 (11.1%)	0 (0%)	1 (8.3%)	9 (6.6%)
Outside Australia	0 (0%)	1 (7.1%)	0 (0%)	0 (0%)	0 (0%)	0 (0%)	0 (0%)	0 (0%)	0 (0%)	1 (0.7%)
**Duration working… (mean years**,** (SD))**										
With individuals with autism	11.7 (± 6.6)	2.8 (± 2.5)	6 (± 6.3)	5.7 (± 5.7)	9.7 (± 9.7)	10.4 (± 13.6)	9.6 (± 9.1)	9.3 (± 6.7)	9 (± 6)	8.3 (± 7.5)
In current role	4.3 (± 4.1)	1.8 (± 2.3)	3.3 (± 4.2)	4.1 (± 5)	6.1 (± 9.3)	1.7 (± 1.2)	4.7 (± 4.5)	3.7 (± 4.2)	4.5 (± 4.4)	4.2 (± 5.3)
In profession	10.8 (± 8.3)	2.5 (± 2.3)	14.9 (± 9.8)	6.8 (± 6.5)	8.4 (± 9.9)	2.7 (± 2.7)	9.9 (± 9.8)	13.5 (± 10.7)	13.4 (± 11.5)	9.9 (± 9.4)

Overall, 136 Australian early intervention providers working directly with children on the autism spectrum participated in the survey. In total, 188 individuals responded to the survey link; of these, 31 (16.5%) exited the survey without providing any responses, leaving 157 participants who provided consent and commenced the survey. Of the latter group, 80 participants (51%) completed at least the demographics section but not the entire survey, and were designated as non-completers. The remaining 77 (49%) completed demographics and stated their preferred approaches for the outcome domains that they targeted; these 77 participants were designated as completers. The survey was formulated around six major subheadings, to be elaborated in detail in the following section. Notably, non-completers typically responded to the first two rubrics (domain priority and confidence), exiting when asked to provide their most common practices for each domain. Apart from the highest education attained, where a higher proportion of non-completers achieved a senior certificate or diploma level qualification compared with completers (*χ*^*2*^(9) = 12.28, *p* =.031), non-completers and completers did not differ on demographics (e.g., gender, age, personal experience, role, and location (all *p* >.05; see Supplementary material - Table A1). The questions could be analyzed separately and sections of the survey did not rely on previous sections. Given that important information could be obtained by analyzing sections separately, the analysis of each section included all participants that addressed it (including non-completers).

### Measures

Participants completed the online survey using the REDCap platform (Harris et al., 2009). In addition to demographics, the survey targeted 10 outcome domains, based on 6 major topics:

(a) provider priorities, (b) provider confidence, (c) practices used for that domain, (d) beliefs about the evidence-base of practices used, (e) interest in PD, and (f) barriers and enablers to empirically supported practice use. The survey was pilot-tested for language and clarity among students of Griffith University and reviewed for sensitivity by the last author and third author, who had previously worked within early intervention services for children on the autism spectrum for 7 years and 5 years, respectively. The feedback from the pilot was incorporated to refine wording and functionality of the survey (e.g., children on the autism spectrum). A copy of the full survey protocol is available from [OSF: https://osf.io/pnqsu/files/osfstorage/67e1d9215e1ef2cfa4449d89].

### Demographics

Questions included age bracket, gender, location of work, highest academic qualification, disability-specific qualifications, place of training, time working with individuals on the autism spectrum, personal experience with children on the autism spectrum, current role, and profession.

### Domains of Practice, Practice Priorities, and Confidence in Practice

We presented participants with 10 outcome domains (adaptive behavior, challenging behavior, communication, joint attention, motor, play, pre-academic, school transition, sensory, social skills) related to early intervention for children on the autism spectrum based on Dynia et al. (2020) adapted from Wong et al. (2015). Each domain was briefly elaborated in one-to-two sentences to ensure uniform understanding. A complete list of domain rubrics and the description of each are available from [OSF: https://osf.io/pnqsu/files/osfstorage/67e1d931b2bfcb337989067e]. For each domain, participants rated the priority of the service and their confidence in addressing the domain. Priority ratings were on a 5-point scale from Not a priority (0) to Very high priority (4). Confidence ratings were also on a 5-point scale: Not at all confident (0) to Very confident (4).

### Practices Used and Evidence-Base

Participants were asked to list their top (up to 3) approaches, strategies, or interventions for addressing each domain, with an N/A option afforded to indicate when they did not provide support in a domain (adapted from Dynia et al., 2020). Applying a procedure used in past research (Paynter et al., 2019), participants indicated the practice they used most frequently and were asked to report their understanding of whether the practice was supported by research evidence of its effectiveness, using a 5-point scale: No evidence (0), Low evidence (1), Moderate evidence (2), Strong evidence (3), Indisputable/established as an empirically supported practice (4), with an ‘I don’t know’ option provided. Approximately 40% of participants either selected their favored approach and did not indicate whether they felt that there was evidence to support it or did not indicate their favored approach but mentioned evidence supporting unspecified approaches.

### Data Coding

Participants’ open-ended responses (i.e., top [up to 3] approaches, strategies, or interventions for addressing each domain with children on the autism spectrum) were categorized by the first and last authors and coded into FIP (practices which are selected to address specific goals or needs) vs. non-FIP (e.g., comprehensive treatment models implemented center-wide or referral to a service). Practices identified as FIPs were then further categorized into empirically supported practices vs. unsupported practices using the NCAEP 2022 listing of practices ([NCAEP]; Steinbrenner et al., 2020). Inter-rater reliability was checked between two raters who blind- coded a randomly selected sample of 20% of all responses, and ratings were compared using Cohen’s kappa (κ). Inter-rater reliability for FIP vs. non-FIP was moderate (78.5%, κ = 57.0%.). This is consistent with the broader review literature which offers differing definitions of FIPs (e.g., Steinbrenner et al., 2020 vs. NAC, 2015), making it difficult to discriminate between focused intervention practices (FIPs) and comprehensive programs. To resolve this issue, the authors developed an operational definition of a “focused intervention” practice as “something you do following a description of how it is done.” The first and last authors then recoded the lists of practices into FIP vs. non-FIP. Next, the third author blind-coded another 20% of responses into FIP vs. non-FIP, resulting in an agreement of 94.6%, κ = 88.8%. Only responses defined as a practice were assigned to the categories of empirically supported and unsupported. Non-FIPs were coded as ‘N/A’ and missing data was coded ‘missing.’ Agreement for empirically supported practices vs. unsupported practices was substantial to almost perfect (Landis & Koch, 1977) (97.1%, κ = 80.4%.). Results were reviewed for consistency, and disagreement resolved through consensus.

### Professional Development

Participants were asked whether and to what extent their training had covered practices to avoid using with children on the spectrum, to be rated on a 5-point scale: None (1), A little (2), Somewhat (3), Quite (4), Very much (covered in detail) (5), with an ‘I don’t know’ option provided. This question was developed by the authors for the present study. Further, drawing from questions used by Brock et al. (2014), participants rated their interest in training in each of the 10 outcome domains, on a 5-point scale, from Not at all interested (0) to Very interested (4). They were also asked to indicate their preferred avenues for training, from a list of 15 items (e.g., center meetings, one-to-one coaching, online training). For each option, participants were asked to rate the likelihood of their participation on a 5-point scale, from Not at all likely (1) to Extremely likely (5).

### Barriers and Enablers To Professional Development

Participants also addressed barriers and enablers to PD (based on Brock et al., 2014). They were presented with a list of 10 factors (e.g., time, location, personnel coverage) and asked to indicate the effect of each on the likelihood of their participation, on a 3-point scale: Less likely to participate (1), Neither more nor less likely to participate (2), More likely to participate (3). Participants were also asked if additional factors would influence participation in training and development and to elaborate if they selected ‘yes.’

### Procedure

This study was conducted with the approval of the Human Research Ethics Committee of Griffith university [ref no: GU 2019/187]. Recruitment was conducted via social media and email between June 2022 and October 2022. Requests were sent to the members of authors’ professional networks and to relevant providers on autism provider lists in Australia. Snowball sampling was also used. Participation was voluntary. Participants were required to read the information sheet and provide informed consent via electronic approval before proceeding to the survey. Participants could stop at any time, and data were collected anonymously. The survey took approximately 20 min to complete.

## Results

### Practice Use

A total of 1,758 open-ended responses regarding the top (up to 3) approaches, strategies, or interventions for addressing each domain with children on the autism spectrum were reported by the 77 participants who completed this section. Of these 52.4% were classified as a FIPs, 40.8% were classified as a non-FIPs, and 6.8% were invalid or missing (e.g., “N/A”). Of the approaches classified as FIPs, 87.7% were empirically supported. The most commonly used practices classified as empirically supported were reinforcement (11.7%), modeling (9.8%), prompting (9.4%), visual supports (6.3%), social skills training (5.9%), functional behavioral assessment (5.9%), and naturalistic interventions (5.2%).

Next, the responses were coded to indicate a provider’s use of (1) only empirically supported practices, (2) empirically supported practices and unsupported practices, or (3) only unsupported practices. These classifications were made across all outcome domains and separately for each domain. Overall, empirically supported practices were used by most providers, with more than one-half (53.3%) listing a combination of empirically supported and unsupported practices, a substantial proportion (45.3%) listing only empirically supported practices, and very few (1.3%) reporting only unsupported practices (Table 2). At the domain level, the proportion of approaches employed by providers that were categorized as FIP was highest in the communication (78.2%), challenging behavior (73.4%) and social skills (61.8%) domains, and lowest in the school transition (36.6%), sensory (43.7%) and pre-academic domains (44.7%). More unsupported practices were nominated for the sensory domain (20.4%) than for other outcome domains.

**Table 2:** The proportions of early intervention providers who reported use of empirically supported, unsupported practices, or a mix of practices for each outcome domain and over all domains for children on the autism spectrum

Domain (*n* = number of valid respondents for domain)	Unsupported practices	Combination of empirically supported and unsupported practices	Empirically supported practices
Adaptive Behavior (*n* = 40)	0.0%	12.5%	87.5%
Challenging Behavior (*n* = 64)	5.2%	10.9%	82.8%
Communication (*n* = 67)	3.9%	14.9%	80.6%
Joint attention (*n* = 51)	6.5%	9.8%	80.4%
Motor (*n* = 38)	2.6%	10.5%	84.2%
Play (*n* = 49)	5.2%	2.0%	89.8%
Pre-academic (*n* = 33)	5.2%	3.0%	84.8%
School transition (*n* = 37)	1.3%	10.8%	86.5%
Sensory (*n* = 38)	20.8%	18.4%	39.5%
Social skills (*n* = 55)	1.3%	16.4%	81.8%
Total (*n* = 75)	1.3%	53.3%	45.3%

### Providers’ Beliefs about the Empirical Support of their Selected Practices

Of the providers who responded to this question (*n* = 65), over 80% rated the empirical support of their favored approach to address the adaptive and challenging behavior and communication domains as high (31.1–40.7%) or indisputable (44.1–51.1%) (see Table 3). Conversely, between 10% and 20% of providers responded that their preferred practices were either unsupported by evidence or had limited evidence. Approximately a third of respondents did not know if their preferred practices addressing the motor (*n* = 22) and sensory (*n* = 24) domains were supported by research evidence, whilst over a quarter were also unsure of evidence supporting approaches for academic, adaptive behavior and joint attention domains. Notably, the evidence ratings did not significantly differ by domain (Friedman χ^2^(9) = 11.5, *p* =.244).

**Table 3:** Early intervention providers’ beliefs about the state of the empirical evidence for the efficacy of their preferred support approach by outcome domain for children on the autism spectrum

Domain (number of providers responding)	No evidence (0)		Low evidence (1)		Moderate evidence (2)		High evidence (3)		Indisputable as empirically supported practice (4)		Median (range*)	
	*n* (%)		*n* (%)		*n* (%)		*n* (%)		*n* (%)		*n* (%)	
Academic (*n* = 46)	2	4.3%	4	8.7%	5	10.9%	18	39.1%	17	37.0%	3	(3–4)
Adaptive behavior (*n* = 45)	2	4.4%	1	2.2%	5	11.1%	14	31.1%	23	51.1%	4	(3–4)
Joint attention (*n* = 46)	0	0.0%	2	4.3%	10	21.7%	21	45.7%	13	28.3%	3	(2–4)
Challenging behavior (*n* = 58)	0	0.0%	2	3.4%	7	12.1%	21	36.2%	28	48.3%	3	(3–4)
Communication (*n* = 59)	0	0.0%	1	1.7%	8	13.6%	24	40.7%	26	44.1%	3	(3–4)
Motor (*n* = 38)	3	7.9%	1	2.6%	7	18.4%	15	39.5%	12	31.6%	3	(2–4)
Play (*n* = 51)	0	0.0%	2	3.9%	18	35.3%	17	33.3%	14	27.5%	3	(2–4)
School (*n* = 49)	1	2.0%	4	8.2%	7	14.3%	17	34.7%	20	40.8%	3	(3–4)
Sensory (*n* = 35)	3	8.6%	4	11.4%	9	25.7%	10	28.6%	9	25.7%	3	(2–4)
Social (*n* = 59)	1	1.7%	2	3.4%	14	23.7%	19	32.2%	23	39.0%	3	(2–4)

Note. There was no significant difference across domains in evidence ratings

Of the 65 providers who completed these questions, those not included above responded to each domain with “I don’t know”

Of the FIPs reported by providers, we classified 87.7% as empirically supported practices and 12.3% as unsupported practices. On average, participants rated empirically supported practices as having a higher level of evidence (*median* = 5, IQR 4–5) than unsupported ones (*median* = 4, IQR 3–4), by a significant margin (*W* = -14.0, *p* <.001). Most providers believed there was high or indisputable evidence (*median* = 4 [indisputable evidence], range: 2 to 4 [moderate to indisputable evidence]) supporting their practice of choice, and very few rated the FIP as having little or no evidence. There was generally no agreement between practitioner ratings of evidence and our classification (when practitioner ratings of evidence were dichotomized, ICC = 0; 95% CI ± 1.24).

### Providers’ Priorities for Outcome Domains

Providers’ priority ratings differed significantly across outcome domains (Friedman χ^2^(9) = 381.4, *p* <.001) (see Table 4). Challenging behavior received the highest priority relative to all domains except adaptive behavior, school transition and social skills (Wilcoxen *post hoc* comparison, *p* <.002). The next highest priority was communication (*p* <.001), followed by adaptive behavior, relative to preacademic, joint attention, communication, motor and social domains (*p* <.005). The domains rated the lowest priority were pre-academic skills (relative to all except motor and sensory, *p* <.0001), motor skills (relative to all except pre-academic and sensory, *p* <.001), and sensory behaviors (relative to adaptive behavior, challenging behavior, communication, school transition and social skills, *p* <.005). Respondents ascribed similar priority to play as to joint attention, sensory and school transition, and to social as to adaptive and challenging behavior. School transition and sensory had similar priority to motor and play.

**Table 4:** Early intervention providers’ perceived priority for outcome domains for children on the autism spectrum (*n* = 137)

Domain	Not a priority (1)		Low priority (2)		Moderate priority (3)		High priority (4)		Very high priority (5)		Median (range*)	
Academic	5	(3.7%)	24	(17.6%)	59	(43.4%)	34	(25.0%)	14	(10.3%)	3	(3–4)
Adaptive behavior	5	(3.7%)	3	(2.2%)	15	(11.0%)	47	(34.6%)	66	(48.5%)	4	(4–5)
Joint attention	2	(1.5%)	5	(3.7%)	34	(25.0%)	57	(41.9%)	38	(27.9%)	4	(3–4)
Challenging behavior	1	(0.7%)	1	(0.7%)	7	(5.1%)	20	(14.7%)	107	(78.7%)	5	(4–5)
Communication	1	(0.7%)	5	(3.7%)	13	(9.6%)	39	(28.7%)	78	(57.4%)	5	(5)
Motor	8	(5.9%)	10	(7.4%)	49	(36.0%)	47	(34.6%)	22	(16.2%)	3	(3–4)
Play	2	(1.5%)	8	(5.9%)	37	(27.2%)	52	(38.2%)	37	(27.2%)	4	(3–5)
School	2	(1.5%)	7	(5.1%)	29	(21.3%)	62	(45.6%)	36	(26.5%)	4	(4–5)
Sensory	6	(4.4%)	14	(10.3%)	42	(30.9%)	43	(31.6%)	31	(22.8%)	4	(3–4)
Social	2	(1.5%)	3	(2.2%)	22	(16.2%)	59	(43.4%)	50	(36.8%)	4	(3–5)

Domains differed in their received priority ratings, Friedman χ^2^(9) = 381.4, *p* <.001

### Provider Confidence

Most respondents were quite confident (39.8%) or very confident (27.8%) in their ability, overall. However, providers’ confidence in their practice differed across the outcome domains (Friedman χ2(9) = 164.1, *p* <.001; see Table 5). Most providers were significantly more confident in social, play and communication than all other domains (post-hoc Wilcoxon signed-rank test, *p* ranged from 0.001 to 0.021), and of the former three, providers were significantly more confident addressing communication compared with the social and play domains (*W* = -2.2, *p* =.025). Conversely, providers were not confident, or only somewhat confident, that their practices addressed motor and sensory domains, compared with all other domains (all *p* <.001). In addition, significant, albeit moderate to weak, positive Spearman’s rank order correlations (ρ) were observed for all domains between practitioner confidence and priority in delivering practices. Thus, providers who reported more confidence in a domain ranked that same domain as higher in priority.

**Table 5:** Early intervention providers’ confidence in their ability to address each outcome domain for children on the autism spectrum (*n* = 133)

Domain	Not at all confident (0)		A little confident (1)		Somewhat confident (2)		Quite confident (3)		Very confident (4)		Median (± IQR)	
Academic	6	(4.5%)	15	(11.3%)	35	(26.3%)	48	(36.1%)	29	(21.8%)	3	(2–3)
Adaptive behavior	7	(5.3%)	12	(9.1%)	28	(21.2%)	44	(33.3%)	41	(31.1%)	3	(2–4)
Joint attention	2	(1.5%)	6	(4.5%)	28	21.1%)	62	(46.6%)	35	(26.3%)	3	(2–4)
Challenging behavior	2	(1.5%)	13	(9.8%)	26	(19.7%)	50	(37.9%)	41	(31.1%)	3	(2–4)
Communication	2	(1.5%)	2	(1.5%)	18	(13.5%)	56	(42.1%)	55	(41.4%)	3	(3–4)
Motor	14	(10.5%)	12	(9.0%)	38	(28.6%)	41	(30.8%)	28	(21.1%)	3	(2–3)
Play	2	(1.5%)	4	(3.0%)	19	(14.3%)	62	(46.6%)	46	(34.6%)	3	(3–4)
School	2	(1.5%)	11	(8.3%)	32	(24.1%)	52	(39.1%)	36	(27.1%)	3	(2–4)
Sensory	8	(6.0%)	26	(19.5%)	40	(30.1%)	41	(30.8%)	18	(13.5%)	2	(1–3)
Social	2	(1.5%)	2	(1.5%)	21	(15.8%)	71	(53.4%)	37	(27.8%)	3	(3–4)

Domains differed in providers’ confidence in their practice ability, Friedman χ^2^(9) = 381.4, *p* <.001

### Training and Development

Providers were asked about the training they received in empirically supported practices and their needs and preferences for further training. The majority (92.3%) of participants reported having a tertiary education and most felt that their training had quite (32.3%) or somewhat (24.6%) addressed practices to avoid using when working with children on the autism spectrum. Fewer participants reported having received either detailed (16.9%) or very little (16.9%) training on practices to avoid, with some reporting no coverage (9.2%).

Providers’ interest in further training differed by domain of practice (Friedman’s χ^2^(9) = 47.5, *p* <.001; see Table 6). While most providers specified that they were extremely interested (Median = 3, range = 2–4) in receiving further training in empirically supported practices for all outcome domains, the most interest was garnered by social skills (compared to pre-academic, motor and sensory, *p* <.0001), challenging behavior (compared to pre-academic and motor *p* <.001), and communication (compared to pre-academic and motor *p* <.003). Providers reported less interest in training in pre-academic (than all other outcome domains except motor, play and sensory *p* <.005) and motor (than challenging behavior, communication, school transition and social skills) domains (*post hoc* Wilcoxon signed-rank tests, *p* <.003).

**Table 6:** Early intervention providers’ interest in receiving training in empirically supported practices related to each outcome domain for children on the autism spectrum (*n* = 63)

Domain	Not at all interested (0)		A little interested (1)		Somewhat interested (2)		Quite interested (3)		Extremely interested (4)		Median (range*)	
Academic	6	9.5%	8	12.7%	12	19.0%	12	19.0%	25	39.7%	3	(2–4)
Adaptive behavior	5	7.9%	5	7.9%	9	14.3%	10	15.9%	34	54.0%	4	(2–4)
Attention	2	3.2%	3	4.8%	13	20.6%	14	22.2%	31	49.2%	3	(2–4)
Challenging behavior	3	4.8%	3	4.8%	7	11.1%	9	14.3%	41	65.1%	4	(3–4)
Communication	2	3.2%	3	4.8%	10	15.9%	12	19.0%	36	57.1%	4	(3–4)
Motor	6	9.5%	8	12.7%	14	22.2%	11	17.5%	24	38.1%	3	(2–4)
Play	2	3.2%	6	9.5%	12	19.0%	11	17.5%	32	50.8%	4	(2–4)
School	2	3.2%	1	1.6%	13	20.6%	14	22.2%	33	52.4%	4	(2–4)
Sensory	4	6.3%	9	14.3%	12	19.0%	8	12.7%	30	47.6%	3	(2–4)
Social	2	3.2%	2	3.2%	6	9.5%	11	17.5%	42	66.7%	4	(3–4)

Statistically significant differences were found in practitioner attitudes towards preferred avenues to receive training (Friedman’s χ^2^(9) = 69.0, *p* <.0001). Most providers responded that they were either extremely likely (*M* = 21.7, *SD* = 8.6) or quite likely (*M* = 18.2, *SD* = 6.6) to access further information and training, regardless of the method of delivery (see Table 7). *Post hoc* comparisons (Wilcoxon signed-rank test) revealed that providers were most likely to access online training, internal training (e.g., PD day) and online evidence-based resources. Also, online training ranked significantly higher in preference compared with journal articles (*p* =.002), PowerPoint, website, printed material and Center-internal training rankings (all *p* <.001). Conversely, providers were least likely (either not at all or with a low likelihood) to make use of center meetings (e.g., info bites; weekly meeting), one-to-one coaching, mentoring or modeling, printed material or Power Point presentations. Center meetings and PowerPoint presentations ranked significantly lower in preference compared with other methods of receiving training.

**Table 7:** Early intervention providers’ avenues for receiving information and training. N.B., ratings from 0 (Not likely) to 5 (Extremely likely) (*n* = 59)

Avenue for receiving training	Not at all likely		A little likely		Somewhat likely		Quite likely		Extremely likely		Median (range*)	
Center meetings	6	(9.7%)	9	(14.5%)	12	(19.4%)	21	(33.9%)	14	(22.6%)	3	(1–3)
One-to-one coaching, mentoring, etc.	4	(6.5%)	6	(9.7%)	10	(16.1%)	18	(29%)	24	(38.7%)	3	(2–4)
External training	1	(1.6%)	4	(6.5%)	7	(11.3%)	28	(45.2%)	22	(35.5%)	3	(3–4)
Online training	1	(1.6%)	2	(3.2%)	9	(14.3%)	16	(25.4%)	35	(55.6%)	4	(3–4)
Printed materials	3	(4.8%)	7	(11.3%)	12	(19.4%)	25	(40.3%)	15	(24.2%)	3	(2–3)
Internal training	5	(8.1%)	1	(1.6%)	6	(9.7%)	17	(27.4%)	33	(53.2%)	4	(3–4)
Informal catchup with colleague	1	(1.6%)	5	(8.2%)	12	(19.7%)	17	(27.9%)	26	(42.6%)	3	(2–4)
Workshop	1	(1.6%)	3	(4.8%)	9	(14.3%)	25	(39.7%)	25	(39.7%)	3	(3–4)
Website	4	(6.5%)	4	(6.5%)	19	(30.6%)	17	(27.4%)	18	(29%)	3	(2–4)
Power point presentation	5	(8.1%)	6	(9.7%)	19	(30.6%)	20	(32.3%)	12	(19.4%)	3	(2–3)
Online evidence-based resources	1	(1.6%)	3	(4.8%)	12	(19.4%)	17	(27.4%)	29	(46.8%)	3	(2–4)
Journal articles	3	(4.8%)	5	(8.1%)	15	(24.2%)	15	(24.2%)	24	(38.7%)	3	(2–4)
Other	5	(33.3%)	1	(6.7%)	3	(20%)	1	(6.7%)	5	(33.3%)	3	(0–4)

For each domain, associations were estimated between practitioners’ interest in further training, and their confidence in practicing in that domain and their perception of evidence supporting their favored approach. Moderate (***ρ*** ≥ 0.3) positive correlations were observed between interest in further training and practitioner confidence for academic, adaptive behavior, motor and sensory domains. Positive correlations (***ρ =*** 0.29 to 0.43) were also found between practitioner interest in further training and their belief that the preferred approach was supported by evidence for pre-academic, joint-attention and school transition.

#### Barriers and Facilitators To Engaging in PD

A statistically significant difference was found in attitudes regarding barriers to participation in training using Friedman’s test (χ^2^(9) = 200.6, *p* <.001). *Post hoc* testing with a Wilcoxon signed-rank test revealed that most providers were likely to participate in training courses offered during center opening hours (66.1%). This stipulation ranked significantly higher than all others (see Table 8), and notably, compared to the timing of training: if it was offered during weekends, school holidays or after school (all *p* <.001). Training that resulted in either continuing education units (58.1%) or TAFE/university course credit (37.1%) also ranked higher than most other fields (*p* <.005). Having to travel large distances (75.8%), interstate (59.7%) or overnight (65.4%) to attend training was identified as the strongest barrier to participation (*p* <.005). Whilst the majority of providers responded that there were no other factors that would have a strong impact on whether or not they would participate in training and development (57.4%), almost 20% identified, in an open-ended response, other factors, including the cost of training, whether the training addressed an emerging issue or priority, and the reputation and experience of presenters.

**Table 8:** Early intervention providers’ barriers associated with participation in training. N.B., ratings from 1 (Less likely) to 3 (More likely) (*n* = 61)

Training delivery	Less likely to participate		Neither more nor less likely to participate		More likely to participate		Median (range*)	
Offered during center opening hours (*n* = 62)	3	(4.8%)	18	(29%)	41	(66.1%)	3	(2–3)
Offered after school (*n* = 62)	16	(25.8%)	33	(53.2%)	13	(21%)	2	(1.5-2)
Offered during weekend (*n* = 62)	28	(45.2%)	24	(38.7%)	10	(16.1%)	2	(1–2)
Offered during holidays (*n* = 62)	25	(40.3%)	28	(45.2%)	9	(14.5%)	2	(1–2)
Results in TAFE/University course credit (*n* = 62)	9	(14.5%)	30	(48.4%)	23	(37.1%)	3	(2–3)
Results in continuing education units (CEUs) (*n* = 62)	6	(9.7%)	20	(32.3%)	36	(58.1%)	1	(2–3)
Involves travel out of state (*n* = 62)	37	(59.7%)	22	(35.5%)	3	(4.8%)	1	(1–2)
Requires overnight travel (*n* = 62)	40	(64.5%)	21	(33.9%)	1	(1.6%)	1	(1–2)
Requires driving more than 100 km one-way (*n* = 62)	47	(75.8%)	12	(19.4%)	3	(4.8%)	1	(1-1.5)
Requires substitute personnel coverage (*n* = 61)	34	(55.7%)	26	(42.6%)	1	(1.6%)	1	(1–2)

## Discussion

The aim of the current study was to understand practices across 10 outcome domains that research has highlighted as important when working with children on the autism spectrum: adaptive behavior, challenging behavior, communication, joint attention, motor, play, pre-academic, school transition, sensory, and social skills. Providers also reported their practice priorities and their understanding of these practices in light of existing research evidence of effectiveness. In addition, providers’ confidence in their abilities across the 10 outcome domains was explored, as well as their PD experiences and preferences across and within each domain, and barriers and facilitators to engaging in PD.

Participants reported using empirically supported practices more often than unsupported ones, consistent with previous research (Dynia et al., 2020; Luskin-Saxby et al., 2023; Paynter et al., 2017, 2021; Paynter & Keen, 2015; Stahmer et al., 2005). Almost all participants reported using at least one empirically supported practice, with over half of these practices among a list of seven most cited practices: reinforcement, modeling, prompting, visual support, social skills training, functional behavioral assessment, and naturalistic interventions. The findings are consistent with past research that identified as the most commonly used empirically supported practices visual supports, modeling, prompting, reinforcement and social narratives (Dynia et al., 2020). Many of these favored practices may be popular because they are relatively easy to implement, do not require expensive resources or training, and can target a range of outcomes in different domains (Dynia et al., 2020; [NCAEP]; Steinbrenner et al., 2020).

Yet, consistent with previous research (Dynia et al., 2020; Luskin-Saxby et al., 2023; Paynter & Keen, 2015; Stahmer et al., 2005), we also found that early intervention providers continue, albeit more rarely, to use practices without research support of their efficacy, either in place of or in addition to empirically supported practices. Most common was the use of unsupported practices to address the sensory domain (e.g., sensory toys such as fidgets, swings, sensory diet)– a finding that aligns with Dynia et al. (2020), where nearly half of the participants reported using sensory-related interventions. The continued use of sensory strategies is perhaps due to their intuitive appeal and because sensory differences are part of the diagnostic criteria for autism (American Psychiatric Association, 2013; Luskin-Saxby et al., 2023). Furthermore, potentially adding to the confusion, sensory interventions are classified differently across reviews. For example, Ayres Sensory Integration Therapy was classified as an empirically supported practice in Steinbrenner et al. (2020), whereas other reviews (e.g., Novak & Honan, 2019) do not categorize any sensory intervention as empirically supported. The scenarios exemplified above highlight the need for more research and consistent methods of classification and communication with reference to the evidence base of interventions targeting sensory processing.

Of the 10 outcome domains examined, participants reported as their highest priorities addressing children’s communication, challenging behavior, adaptive behavior, and social skills, in that order. Past research has also found that top-priority domains are social skills, communication, and challenging behavior (Dynia et al., 2020). Furthermore, most providers in the current study reported that practices they favored to address the communication and challenging behavior domains were empirically supported. This finding accords with those of both Dynia et al. (2020) and Brock et al. (2014), who found that participants’ belief they were using empirically supported practices was significantly predictive of whether empirically supported practice use was coded by the researchers. The results presented above may both reflect and affect the practices early intervention providers use in their work, as the outcome domains these providers prioritize may differ in terms of the number of empirically supported practices available (e.g., more for challenging behavior compared to play; [NCAEP]; Steinbrenner et al., 2020). Moreover, practices may vary in terms of the resources and training required for their implementation: for example, no specialist training is needed for reinforcement, while PECS calls for training as well as resources (e.g., see costs for PECS training: https://pecsaustralia.com/). Finally, only few empirically supported practices have received endorsement specifically for use with very young children (Steinbrenner et al., 2020).

Domains cited in the current study as the lowest priority were academic, motor, and sensory, a result which supports and extends past research (Dynia et al., 2020). This priority placement is noteworthy as children on the spectrum often under-perform academically (e.g., see Keen et al., 2016). Thus, the pre-academic domain should arguably be an important focus for early learning services, to prepare children for subsequent education. It may be, however, that challenging behavior and difficulties with communication are more salient for early intervention providers, while academic difficulties are an issue often seen as crucial for school-age children, who face academic demands (Sulek et al., 2024). Early intervention providers may also see academics as being outside of their area of focus or specialty.

Providers’ confidence across outcome domains also varied. Providers expressed rather more confidence in addressing the domains of communication and play. However, in the domains of sensory, motor, and adaptive behavior, an overwhelming proportion of participants reported feeling little to no confidence. In line with Dynia et al. (2020), this study has yielded evidence that providers’ confidence level regarding the evidence base of early intervention practices for children on the autism spectrum may be domain specific. It is higher in some domains, e.g., communication (Dynia et al., 2020), but in others– notably, sensory– providers may not have a good grasp on which practices are empirically supported and which are not, thereby impacting autism early intervention at the direct service level and exacerbating the research-to-practice gap in this field. In addition, providers’ priorities and their confidence levels were positively correlated, such that providers ascribed higher priority to the domains that they felt more confident in addressing.

Most participants felt that the training they had received did, to a varying degree, address practices to avoid when working with children on the autism spectrum. A similar proportion of providers felt that they had received either detailed training or very limited training on practices to avoid. These results differ from Paynter et al. (2019) and from Luskin-Saxby et al. (2023), in which providers reported that the PD they had received did not specify *what not to do*. However, the finding that some empirically unsupported practices were incorrectly rated as empirically supported suggests that further training may still be required in *what not to do.* An additional factor that may cause confusion is that research tends to convey contradictory messages. For example, sensory integration is listed as an “Ineffective/Don’t Do It” autism intervention in Novak and Honan (2019), and a specific type of sensory integration therapy (Ayres Sensory Integration Therapy) is listed as an EBP in Steinbrenner et al. (2020). More research is needed to address the effectiveness of potentially promising practices.

Most providers reported an interest in receiving further PD and training in empirically supported practices for all outcome domains, regardless of the format in which these services are provided. This contrasts with findings of Brock et al. (2014), whereby respondents expressed a modest interest in accessing additional PD. At the same time, most participants of the current study were especially interested to learn more about social skills, challenging behavior and communication. These preferences are not surprising, as both in the current study and in Dynia et al. (2020), the majority of participants cited these outcome domains as top priorities in terms of outcomes. Differences between countries (e.g., U.S.A.: Brock et al., 2014) may be due to variations in systems, in knowledge of autism, or perhaps in ways of framing survey questions.

Regarding the method through which participants preferred PD and training to be delivered, the majority reported a preference for online training, internal training (e.g., PD day) and online empirically supported resources. The least desired were center meetings, one-to-one coaching, mentoring, or modeling. The lack of inclination revealed for the latter three formats is in contradiction to many past studies (e.g., Trembath et al., 2015, 2019; Keen et al., 2017; Luskin-Saxby et al., 2023), but in line with Brock et al. (2014). In this connection, research has noted some practical difficulties associated with in situ PD and training. For one, this format requires extensive PD, ongoing coaching, and significant time to ensure PD and training are conducted with fidelity. Moreover, to yield improvements in educators’ instructional capacity and in outcomes for children on the autism spectrum, this type of PD must be of high quality and intensity, which may not be readily available (e.g., Brock et al., 2014; Howlin et al., 2007; Odom et al., 2013). The scarcity of effective PD and training may pose barriers in translating research to practice (Brock et al., 2014; Stahmer et al., 2015). The preference participants expressed for online training and online empirically supported resources could be accounted for by the circumstances of the recent COVID-19 pandemic, during which people became accustomed to working from home, away from other people. It stands to reason that, before the pandemic, preferences might have been different. More research needs to be conducted into the impact of online training post-COVID-19. An important question to address would be whether accessing information on one’s own, through online training modules, might have an impact on practice (cf. Ruble et al., 2013).

The correlation between interest in receiving training and confidence in using empirically supported practices for academic, adaptive behavior, motor and sensory domains was moderately positive. Those with more confidence were moderately more interested in receiving further training. However, this finding differs from Brock et al. (2014), who found no relation between participants’ interest in receiving training and their confidence in that regard. Furthermore, providers’ belief that they were using empirically supported practices was not significantly related to their eagerness to obtain PD– in line with Dynia et al. (2020), who found no significant relation between a teacher’s confidence or belief they were using empirically supported practices and their eagerness to obtain PD. In this regard, too, differences between countries may be due to differing training programs or understanding of autism and of empirically supported practices.

Most providers responded that they were most likely to participate in training courses if these were conducted during the center’s opening hours. This finding corroborates previous research revealing time to be a significant barrier to PD and training (Barry et al., 2020; Brock et al., 2014). Furthermore, participants in the current study sought recognition for their training as either continuing education units or TAFE/university course credit. Consistent with previous research (Luskin-Saxby et al., 2023), providers reported the least likelihood to attend if the PD was interstate or required overnight stay. This attitude had also emerged in previous research conducted in Australia, and is quite understandable, as the geographic spread of the country can militate against accessing support, particularly in the rural and remote areas, which are vast (Luskin-Saxby et al., 2023). Factors found to affect the likelihood of participation in training align with interest providers expressed in online training, which may mitigate this barrier.

Few participants provided feedback on other factors which might impact their decision to participate in further PD. Among these was the cost of training– a finding that dovetails with those of previous research indicating that resources can be a significant barrier to PD (Brock et al., 2014; Luskin-Saxby et al., 2023; Paynter et al., 2017; Paynter & Keen, 2015). Other such factors included whether the training addressed an emerging issue or priority, and the reputation and experience of presenters. These results relate to a significant barrier to PD found in previous research: reliance on anecdotal sources of information, notably, colleagues’ reports (Paynter et al., 2018)– a factor that has been linked to gaps in knowledge, to false beliefs about the evidence base of different practices (e.g., Kadar et al., 2012; Miller et al., 2012; Paynter et al., 2018), and to a lack of PD (Dillenburger et al., 2016; Elsabbagh et al., 2014). Another notable barrier to PD that emerged in this study was related to the issue of whether the PD content is supported and endorsed by the autistic community: Providers emphasized interest in hearing from people with lived experience, an attitude which accords with recent movements in the field (Sulek et al., 2024).

### Limitations and Future Research

The present study has some limitations that should be acknowledged. First, results should be interpreted with reference to the settings within which the data were collected, that is, services in Australia. Further, this study looked narrowly at the evidence-base of the various practices and not at the broader EBP framework. Future research into clinical decision-making should probe whether choices are consistent with family needs, are influenced by a lack of available empirically supported practices in some outcome domains, and are optimal considering the resources, training, and practices available. This study’s substantive strength is in allowing providers to list practices or strategies, and rate their level of empirical evidence of effectiveness, rather than providing lists of practices to tick or confirm, which could bias results via acquiescence. While this approach contributed to the study’s validity, survey completion presented a measure of difficulty for some providers. Possibly due to the complexity of some questions, the drop-out rate in survey completion was higher than desired. Future research may opt to reimburse participants for time, and to partner with organizations to embed in practice (e.g., to audit organization-wide as a quality assurance mechanism), in order to make larger, more representative samples more feasible, which in turn would boost exploration and inform changes in specific contexts or settings.

### Conclusions and Implications

The current study has demonstrated that early intervention providers working with children on the autism spectrum predominantly use empirically supported practices, particularly those aimed at improving communication skills. However, they also tend to incorporate practices that are not empirically supported, especially those targeting sensory-related issues. The ratio is contingent on the specific outcome domain and on the priority providers assign to areas they deem most important in their work or in which they feel most confident. The link that emerged between confidence and domain priority, as well as the limited number of empirically supported practices that providers reported using, suggests a need for broader PD and training encompassing all outcome domains as well as practices to avoid (*what not to do*).

The study has also pointed to a number of challenges to the across-the-board use of empirically supported practices, including constraints in terms of time and resources, as well as reliance on anecdotal information to ground decision-making. These drawbacks were found to extend the PD that is currently available to early intervention providers. The results obtained here can inform PD in early intervention services for children on the autism spectrum. One example is a “train-the-trainer” resource package (such as used in the U.S.A., Ruble et al., 2013), based on state-of-the art research on empirically supported practices for children on the autism spectrum (NAC, 2015; Steinbrenner et al., 2020). Research in this direction would facilitate greater opportunities for all children on the spectrum to receive the most efficacious supports to achieve the best possible outcomes. It would also help expedite the translation of knowledge to practice and bridge the research-to-practice gap in autism early intervention, thereby ensuring optimal early intervention and best possible outcomes for children on the autism spectrum.

## Electronic supplementary material

Below is the link to the electronic supplementary material.

Supplementary Material 1

## Footnotes

1 Used henceforth as the most commonly preferred term to describe autism; informed by Australian research by Bury et al., 2023.
