# Supplementary material for: Autism Early Intervention Providers: Their Priorities, Use of Empirically Supported Practices, and Professional Development Needs
Source: J Autism Dev Disord. 2025 Apr 3;56(9):3343–58. doi: 10.1007/s10803-025-06808-w (PMC13427875; doi:10.1007/s10803-025-06808-w)
Supplement: Supplementary file 1 — Supplementary Material 1 [file 10803_2025_6808_MOESM1_ESM.docx]

Table A1: Demographics of all respondents completing at least the demographics section and those who completed the whole survey (unless otherwise stated, n (%))

| **Participant characteristic** | **Incomplete**  **(n = 80)** | | **Complete**  **(n = 77)** | | **Total**  **(n = 157)** | |
| --- | --- | --- | --- | --- | --- | --- |
| **Sex** |  |  |  |  |  |  |
| Male | 2 | (2.5%) | 5 | (6.5%) | 7 | (4.5%) |
| Female | 78 | (97.5%) | 71 | (92.2%) | 149 | (94.9%) |
| Non-binary | 0 | (0%) | 1 | (1.3%) | 1 | (0.6%) |
| **Age bracket** |  |  |  |  |  |  |
| Under 25 | 18 | (22.5%) | 18 | (23.4%) | 36 | (22.9%) |
| 26-35 | 26 | (32.5%) | 25 | (32.5%) | 51 | (32.5%) |
| 36-45 | 19 | (23.8%) | 15 | (19.5%) | 34 | (21.7%) |
| 46-55 | 12 | (15%) | 13 | (16.9%) | 25 | (15.9%) |
| 56-65 | 5 | (6.3%) | 4 | (5.2%) | 9 | (5.7%) |
| 66 and over | 0 | (0%) | 2 | (2.6%) | 2 | (1.3%) |
| **Highest academic qualifications*** |  |  |  |  |  |  |
| Grade 9 of High School or less | 1 | (1.3%) | 0 | (0%) | 1 | (0.6%) |
| Junior Certificate | 0 | (0%) | 0 | (0%) | 0 | (0%) |
| Higher school certificate | 8 | (10%) | 1 | (1.3%) | 9 | (5.7%) |
| Diploma or equivalent | 11 | (13.8%) | 6 | (7.8%) | 17 | (10.8%) |
| Bachelor’s degree | 24 | (30%) | 21 | (27.3%) | 45 | (28.7%) |
| Postgraduate degree | 36 | (45%) | 46 | (59.7%) | 82 | (52.2%) |
| Other | 0 | (0%) | 3 | (3.9%) | 3 | (1.9%) |
| **Location of training** |  |  |  |  |  |  |
| In Australia | 64 | (81%) | 61 | (79.2%) | 125 | (80.1%) |
| Overseas | 8 | (10.1%) | 6 | (7.8%) | 14 | (9%) |
| Both | 7 | (8.9%) | 10 | (13%) | 17 | (10.9%) |
| **Personal experience with children on the autism spectrum** | | | | | | |
| Yes | 29 | (36.7%) | 36 | (46.8%) | 65 | (41.7%) |
| No | 50 | (63.3%) | 41 | (53.2%) | 91 | (58.3%) |
| **Role** |  |  |  |  |  |  |
| Behaviour analyst | 12 | (15.2%) | 17 | (22.1%) | 29 | (18.6%) |
| Behaviour therapist | 9 | (11.4%) | 6 | (7.8%) | 15 | (9.6%) |
| Educator or learning facilitator | 19 | (24.1%) | 8 | (10.4%) | 27 | (17.3%) |
| Occupational therapist | 6 | (7.6%) | 9 | (11.7%) | 15 | (9.6%) |
| Provisional psychologist | 3 | (3.8%) | 6 | (7.8%) | 9 | (5.8%) |
| Psychologist | 6 | (7.6%) | 10 | (13%) | 16 | (10.3%) |
| Social worker | 1 | (1.3%) | 1 | (1.3%) | 2 | (1.3%) |
| Speech pathologist | 8 | (10.1%) | 11 | (14.3%) | 19 | (12.2%) |
| Teacher | 5 | (6.3%) | 3 | (3.9%) | 8 | (5.1%) |
| Other | 10 | (12.7%) | 6 | (7.8%) | 16 | (10.3%) |
| **Duration (mean (±SD))** |  |  |  |  |  |  |
| in role | 3.4 | (±4.2) | 4.7 | (±6.2) | 4.1 | (±5.27 |
| in profession | 10.4 | (±9.6) | 9.4 | (±9.9) | 10.2 | (±9.73) |
